# Mott Cells in the Peripheral Blood of a Patient with Dengue Fever

**DOI:** 10.4274/tjh.2015.0082

**Published:** 2015-12-03

**Authors:** Aniya Antony, Marie Ambroise, Chokka Kiran, Mookkappan Sudhagar, Anita Ramdas

**Affiliations:** 1 Pondicherry Institute of Medical Sciences, Clinic of Pathology, Puducherry, India; 2 Pondicherry Institute of Medical Sciences, Clinic of General Medicine, Puducherry, India

**Keywords:** Infection, Platelets, Lymphocytes, Viral infection

## IMAGE IN HEMATOLOGY

**Figure 1:**
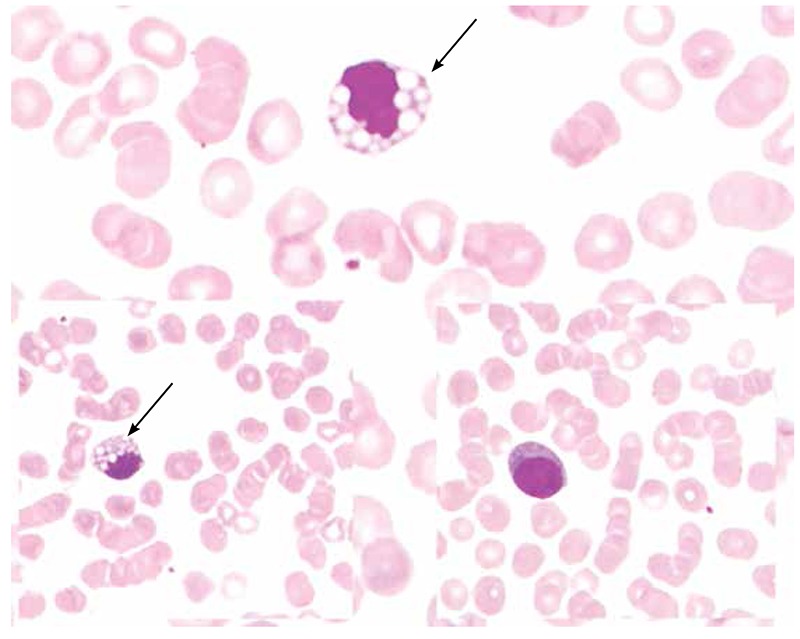
The top image shows a Mott cell. The bottom left image shows a similar Mott cell packed with spherical cytoplasmic inclusions. The bottom right image shows a plasmacytoid lymphocyte with deep basophilic cytoplasm (Leishman stain; magnification 1000x).

A 48-year-old female presented with intermittent high-grade fever, chills, and severe myalgia for 4 days. There was no lymphadenopathy or hepatosplenomegaly. Investigations revealed hemoglobin concentration of 142 g/L; leucocyte count of 3.5x109/L with 54% neutrophils, 40% lymphocytes, 1% eosinophils, and 5% monocytes; and thrombocytopenia (platelet count of 55x109/L). Peripheral smear revealed numerous plasmacytoid lymphocytes and occasional cells with eccentrically placed nucleipacked with multiple prominent cytoplasmic vacuoles, morphologically consistent with Mott cells (Figure 1). Meanwhile, Dengue NS1 antigen assay turned out to be positive. The patient was managed conservatively and discharged after 4 days with a platelet count of 150x109/L. Peripheral smear revealed only occasional reactive lymphocytes and the Mott cells had disappeared. Three weeks after discharge, platelet and leucocyte counts had improved further.

Nonmalignant reactive peripheral blood plasmacytosis can occur in tumors, autoimmune conditions, and infections [1]. Polyclonal peripheral blood plasmacytosis also occurs in Dengue virus infections and is prominent during the first week of the disease [2]. However, the transient occurrence of Mott cells in the peripheral blood of Dengue fever patients has not been reported previously. Our patient was not suffering from lymphoma or multiple myeloma, which are potential causes of peripherally circulating Mott cells.
